# Wireless Tri-Axial Trunk Accelerometry Detects Deviations in Dynamic Center of Mass Motion Due to Running-Induced Fatigue

**DOI:** 10.1371/journal.pone.0141957

**Published:** 2015-10-30

**Authors:** Kurt H. Schütte, Ellen A. Maas, Vasileios Exadaktylos, Daniel Berckmans, Rachel E. Venter, Benedicte Vanwanseele

**Affiliations:** 1 Human Movement Biomechanics Research Group, Department of Kinesiology, KU Leuven, Leuven, Belgium; 2 Measure, Model & Manage Bioresponses (M3-BIORES) Group, Department of Biosystems, KU Leuven, Leuven, Belgium; 3 Movement Laboratory, Department of Sport Science, Stellenbosch University, Stellenbosch, Western Cape, South Africa; St Francis Hospital, UNITED STATES

## Abstract

Small wireless trunk accelerometers have become a popular approach to unobtrusively quantify human locomotion and provide insights into both gait rehabilitation and sports performance. However, limited evidence exists as to which trunk accelerometry measures are suitable for the purpose of detecting movement compensations while running, and specifically in response to fatigue. The aim of this study was therefore to detect deviations in the dynamic center of mass (CoM) motion due to running-induced fatigue using tri-axial trunk accelerometry. Twenty runners aged 18–25 years completed an indoor treadmill running protocol to volitional exhaustion at speeds equivalent to their 3.2 km time trial performance. The following dependent measures were extracted from tri-axial trunk accelerations of 20 running steps before and after the treadmill fatigue protocol: the tri-axial ratio of acceleration root mean square (RMS) to the resultant vector RMS, step and stride regularity (autocorrelation procedure), and sample entropy. Running-induced fatigue increased mediolateral and anteroposterior ratios of acceleration RMS (p < .05), decreased the anteroposterior step regularity (p < .05), and increased the anteroposterior sample entropy (p < .05) of trunk accelerometry patterns. Our findings indicate that treadmill running-induced fatigue might reveal itself in a greater contribution of variability in horizontal plane trunk accelerations, with anteroposterior trunk accelerations that are less regular from step-to-step and are less predictable. It appears that trunk accelerometry parameters can be used to detect deviations in dynamic CoM motion induced by treadmill running fatigue, yet it is unknown how robust or generalizable these parameters are to outdoor running environments.

## Introduction

Given the repetitive nature of running, it is generally recommended that a running bout be stopped at a point before an individual’s typical level of fatigue causes adverse biomechanical effects and subsequent injury [1]. Indeed, runners in a fatigued state have shown to lose coordination and consistency over positioning of their lower extremities [2–4] and become less capable at attenuating tibial shock accelerations [5]. Identifying and correcting deterioration in a runner’s technique due to fatigue is of value to enhancing athletic performance and might have potential to prevent injuries [6].

In contrast to the lower extremity which has been well studied [5,7,8], the response of the trunk or whole body center of mass (CoM) to running-induced fatigue has received limited attention. The trunk serves a number of functions beneficial during locomotion, such as to attenuate accelerations that reach the head [9] or to maintain upright posture [10]. Koblbauer et al., [11] showed for novice runners, that besides an increase in peak ankle eversion angles, the most pronounced compensation in kinematics due to running-induced fatigue was an increase in forward inclination of the trunk. Morin et al., [3] reported that along with increased leg stiffness, the vertical motion of the CoM significantly reduced with prolonged exhaustive running. Reducing energetic cost [12–14], self-preservation of musculoskeletal structures [3], and pain avoidance [3] have all been considered mechanisms to explain the deviations in CoM motion during running.

In comparison to traditional 3D motion capture methods, a single tri-axial trunk accelerometer offers a valid [15–17] and reliable [18,19] approach to measure trunk and CoM acceleration during human locomotion and facilitates unobtrusive data collection in various environments. Trunk accelerometry measures of human gait that have shown to objectively detect deviations in dynamic CoM motion include: variability, represented as the root mean square (RMS) of accelerations contributing to each independent axis [19,20], step and stride regularity, assessed by the unbiased autocorrelation procedure [18,21–23], and the sample entropy value [24]. The latter is a non-linear statistic that considers the complexity of movement variability which may be masked or ignored using traditional measures [25].

Although CoM accelerometry measures have been well established for assessing walking gait [20,21,23,26,27], limited investigations exist for assessing running gait [4,19,28]. Only two studies [4,19] have specifically used trunk accelerometry measures to assess running-related fatigue. The former study [4] found a decrease in regularity of vertical CoM accelerations, and an increase in the impulse of mediolateral CoM accelerations when their sub-elite distance runners underwent a short but highly intensive track run to exhaustion. The latter study [19] showed that increases in vector RMS of trunk accelerations could accurately estimate increases in metabolic work (VO_2_) during an incremental running protocol to exhaustion. However, they [19] additionally reported that the vector RMS of trunk accelerations was associated with increments in running speed. Thus, albeit large potential, limited evidence exists [4] as to which trunk accelerometry measures are suitable for the purpose of detecting compensations in dynamic CoM motion while running under stead-state conditions. Moreover, measures such as sample entropy that consider the complexity of movement patterns may reveal additional insights into CoM fatigue-related compensations that have previously been unexplored.

The aim of this study was to detect deviations in dynamic CoM motion in relation to running-induced fatigue using tri-axial accelerometry. We hypothesized that when runners were in a fatigued state, a single trunk–mounted accelerometer would be able to detect changes primarily occurring in the horizontal plane. More specifically, we expected mediolateral and anteroposterior trunk acceleration patterns of fatigued runners to demonstrate 1) a greater contribution of variability (larger ratio of acceleration RMS), 2) less step and stride regularity, and 3) less predictability (higher sample entropy values).

## Methods

### Participants

Twenty-two runners aged 18–25 years were recruited to participate in this study. Participants were recruited via social media, e-mails and flyers. Runners were eligible for the study if they ran at least 10 km per week and ranged in experience from novice to competitive. Exclusion criteria were pulmonary, neurological and cardiovascular diseases, muscle weakness and obesity, assessed through a questionnaire prior to study participation. Runners with overuse injuries in the previous six months were excluded, as well as runners with orthopedic devices, except insoles. The study was approved by the local ethics committee (Commissie Medische Ethiek KU Leuven). Written informed consent was signed by participants or on behalf of a legal guardian prior to study participation in accordance with the Declaration of Helsinki.

### Experimental protocol

On day one the participants performed a 3.2 km run at maximal effort on an outdoor track. The time was recorded to determine their average running speed to be subsequently used for the treadmill fatigue protocol. On day two, participants completed an exhaustive treadmill run, set at speeds equivalent to their 3.2 km time-trial track performance. The aim of the exhaustion protocol was to have participants run to voluntary exhaustion, or when the termination criteria was achieved (BORG-score of at least 17/20 [29]). There were a minimum of 7-and a maximum of 10-days between testing day one and two [30]. Participants were instructed to abstain from arduous exercise for the 24 hours prior to both testing days, and to maintain their regular running schedule between tests. Each participant had previous treadmill running experience. On testing day two participants were given at least five minutes to acclimatize to the motorized treadmill of the laboratory where data collection took place. This also served as their warm-up. Some participants had time-trial speeds that exceeded the maximum speed of the laboratory instrumented treadmill (3.33 m/s). For these participants, pre-fatigue and fatigued conditions were fixed at 3.33 m/s, and they had to perform the exhaustive run on a separate motorized treadmill that enabled time-trial speeds. Due to logistical reasons, this separate treadmill could not be placed within the capture volume of the motion analysis system, and thus sacral marker trajectory could not be recorded during the fatigue protocol. Since we felt it was imperative that participants’ were fatigued at their time-trial speed, the primary scope of this study was delimited to a pre-post fatigue design. The *pre-fatigue* condition was defined as the first two minutes of the fatigue protocol, and the last 10-seconds of this period was extracted for processing (three dimensional motion analysis and trunk accelerometry measurements). The fatigued condition was defined as the last 10-seconds prior to the aforementioned fatigue-related termination criteria. For participants that were fatigued on a separate treadmill; their fatigued condition was defined as the last 10-seconds of the first minute upon re-transitioning to the laboratory treadmill. The post-fatigue period between treadmill transitioning was never more than 30 seconds. Standardized running shoes were provided for all participants (Asics Gel Landreth 7).

### Three dimensional motion analysis

A ten camera Vicon system (Vicon®, Oxford, Metrics UK) was used to track the motion of the retro-reflective sacral marker sampled at 150 HZ. The *sacral marker displacement method* was used to represent the total body CoM displacement, a method that has shown to be accurate during walking [31] and running [32]. Three dimensional marker trajectory was recorded during the last 10 seconds of each test condition. Trials were deemed to be successful if at least 20 consecutive steps of sacral marker visibility could be obtained.

### Accelerometry measurements

A single tri-axial accelerometer (X16-2 wireless accelerometer, range ±16g, resolution 15-bit, Gulf Coast Data Concepts, MS) sampling at 400 Hz and weighing 48 g was mounted over the L3-L5 spinous process of each runner with double-sided tape [18]. Additional elastic straps were used to secure the accelerometer and minimize unnecessary movement. Trials were discarded in the case that the investigators deemed the accelerometer to be “not securely fastened” upon its removal (after completion of the data collection). Trunk accelerometry signals were recorded throughout the pre-fatigue and fatigue conditions (on-board SD card).

### Data reduction

All data processing and analyses were performed using customized MATLAB software version 8.3 (The Mathworks Inc., Natick, MA, USA). Dependent variables assessed in this study were derived from 3D sacral marker trajectories (mean displacement and range) and tri-axial trunk accelerations (acceleration RMS, ratio of acceleration RMS, step regularity, stride regularity, and sample entropy). From 10-seconds of signals (trajectories and accelerations), the first 20 running steps were extracted to calculate each dependent variable.

Vertical, mediolateral, and anteroposterior sacral marker trajectories were filtered using a zero-lag 4th order low-pass Butterworth filter with a cut-off frequency of 15 Hz. Vertical displacement was determined by averaging the peak-to-peak difference (maximum-minimum) [31] of exactly 20 consecutive running steps for each participant. Since anteroposterior and mediolateral trajectories depend to some extent on where the participant is positioned on the treadmill, the start and end points for each step are not the same (each step thus results in a net gain or loss in displacement), rendering step-to-step displacements for these directions not as useful [33]. Rather, we used the procedure devised by Hinrichs et al., [33] to calculate anteroposterior and mediolateral displacement: by computing the total excursion per step (sum of the absolute displacements between samples (frames), and finally averaging over 20 steps. Steps were identified according to the locations of the peaks in the vertical trajectory. Additionally, we calculated the range, which was the maximum distance (absolute maximum-minimum) between any two points of each axis over the 20-step interval.

The raw vertical, mediolateral, and anteroposterior signals from the accelerometer were converted from counts to g’s offline, trigonometrically corrected to remove the static gravitational component [18], and filtered using a zero-lag 4th order low-pass Butterworth filter with a cut-off frequency of 50 Hz. The sensing axis of the accelerometer may not be aligned with the axes of the horizontal-vertical coordinate system of the laboratory while running. Therefore, a trigonometric correction [18] of the acceleration signal was performed, a procedure consistently applied to CoM accelerations during walking [10,18,20] and running [16,19,28]. In this study, calculated deviations of accelerometer axes were between 3.5 degrees to 9.3 degrees (anterior tilt) and 0.1 to 1.2 degrees (laterolateral tilt) prior to axis transformation. This procedure also enabled accelerometer alignment with the axes of the sacral marker. Tri-axial trunk accelerometry measures were examined using the acceleration root mean square (RMS), the RMS ratio of each axis to the resultant vector [19], step regularity and stride regularity [18], and sample entropy [25] of accelerations.

The acceleration root mean square (RMS) was calculated for each axis independently and gives an overall indication of variability of acceleration dispersion [18,19]. Next, the acceleration RMS ratio, an indicator of the proportion of accelerations in each axis contributing to the overall movement, was calculated as the RMS of each axis relative to the resultant vector RMS [19,20].

**Fig 1:**
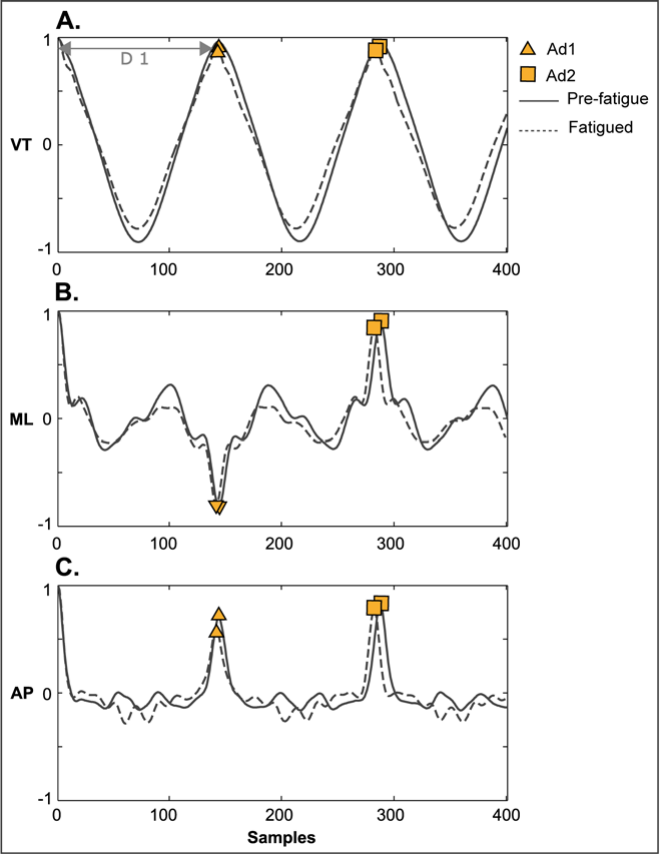
Unbiased autocorrelation patterns derived from vertical (A), mediolateral (B), and anteroposterior (C) acceleration signals of one representative subject running pre-fatigue (solid line) and fatigued (dashed line). The first (Ad1) and second (Ad2) dominant autocorrelation peaks represent step and stride regularity measures respectively. The distance (D1, grey arrow) from time zero shift to Ad1 in the vertical pattern is used to calculate step frequency.

Step and stride regularity of accelerations were computed using the unbiased autocorrelation procedure previously described by Moe Nilssen et al., [18]. Representative unbiased autocorrelation patterns of all three acceleration axes are shown in **Fig 1**. Step regularity, the first dominant autocorrelation peak (Ad1 in **Fig 1**), indicates a correlation between consecutive steps and is therefore considered the symmetry index. Since mediolateral trunk accelerations produce both positive and negative accelerations that represent left–to-right lateral trunk motion, step regularity values for the mediolateral direction are always negative (Ad1 in **Fig 1B**). The absolute value for mediolateral step regularity was therefore used for analysis. Step frequency was computed from the vertical axis of the sequence of trunk accelerations using samples per dominant period of the autocorrelation peak and sampling frequency of the accelerometer as inputs [18] (D1 in **Fig 1A**). Stride regularity (Ad2 in **Fig 1**), the second dominant autocorrelation peak, represents a correlation between consecutive strides and can be considered as a regularity index. After normalization to the zero lag component, the maximum value (most periodic, most regular) for both step regularity and stride regularity is one.

Lastly, the sample entropy of accelerations was determined using the non-linear mathematical algorithms previously described in detail by Richman and Moorman [25] and quantifies the uncertainty or unpredictability of the accelerometry time series [34], with a larger value indicating a less periodic and less predictable or periodic pattern. In contrast to the aforementioned measures, sample entropy was analyzed from unfiltered accelerations so as not to mask or remove any dynamical properties or variability present within the system [25]. In the literature, there are two contrasting approaches in human gait analysis to select the data string length parameter for sample entropy, either according to a fixed number of samples (time) [35,36], or according to a fixed number of gait cycles [37]. In contrast to its predecessor statistic (approximate entropy), sample entropy values are more robust to shorter data strings and become stable at data strings exceeding over 2000 samples [34]–all of our trials were beyond this length to acquire 20 consecutive running steps (minimum was 2700 samples). Thus, we selected the “fixed-step” approach, also enabling consistency in number of steps selected from the sacral marker trajectory. Therefore, input parameters for our sample entropy calculation were firstly, a time series sample length (N) equivalent to 20 running steps (typical data string between 2700 to 3300 data points), secondly, a series length (m) of 2 data points, and thirdly, a tolerance window (r) normalized to 0.2 times the standard deviation of individual time series [34].

### Statistical analysis

All statistical analyses were performed using SPSS (version 20.0; SPSS Inc, Chicago, IL). Descriptive statistics were computed for all participant characteristics. Changes between pre-and post-fatigued dependent measures were assessed using repeated measures ANOVA. All dependent variables that did not meet assumptions for normality (Kolmogorov-Smirnov test with Lilliefors significance correction) were transformed by taking their common based 10 log prior to statistical analysis. If a measure did not achieve normality after log transformation, a non-parametric Friedman’s analysis was performed rather than the repeated measures ANOVA. The level of statistical significance for all tests was set at a value of 0.05.

## Results

Two participants were excluded from analysis due to faulty equipment: one participant was excluded because the sacral marker lost visibility during recording of the fatigued condition (~ 12 complete steps could be identified); and the other participant was excluded since the investigators deemed the accelerometer to be “not securely fastened” upon its removal after data collection. For the latter participant, visual inspection of the vertical axis also revealed clipping (saturation) of peak accelerations beyond the 16 g threshold. The mean [range] of participant characteristics from the remaining 20 participants are represented in **Table 1**. Participants completed their treadmill exhaustion protocol in 20.54 (6.90) minutes and all achieved Borg RPE ratings greater than 17/20 at time of termination.

**Table 1:** Subject characteristics for N = 20 (12 males): mean, (SD), [range].

Age (years)	Height (m)	Weight (kg)	Training volume (km·wk^-1^)	3.2 km time trial speed (m·s^-1^)
21.05 (2.14)[17–25]	1.77 (0.08)[1.61–1.90]	66.12 (6.19)[56.4–74.9]	48.28 (36.18)[10–110]	3.89 (1.24)[2.03–5.69]

**Fig 2:**
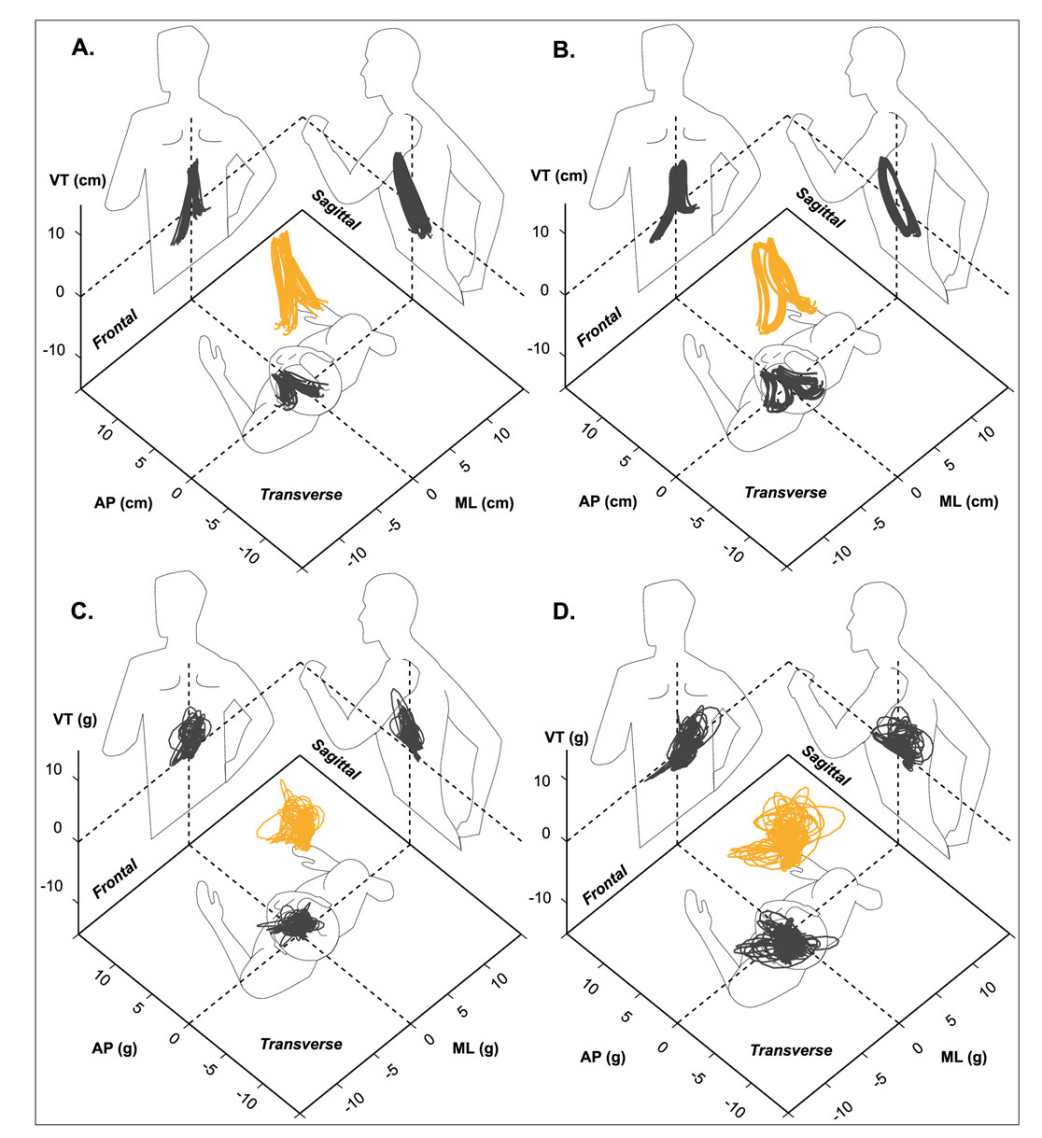
Sacral trajectory (A: pre-fatigue; B: fatigued) and trunk accelerations (C: pre-fatigue; D: fatigued) for 20 consecutive steps of treadmill running of one representative participant. Vertical (VT), anteroposterior (AP), and mediolateral (ML) axes are used to compute three dimensional (in orange) and two dimensional (in grey) projections of CoM motion relative to the planes of movement. The outline of the human body is shown only for the purpose of indicating direction of running (positive AP).

The effect of running fatigue on sacral marker trajectory variables can be seen in **Table 2**. In the fatigued condition, runners significantly increased their CoM displacements and range for mediolateral and anteroposterior directions, but not for the vertical direction. An exemplary stabilogram of changes in horizontal sacral marker trajectory for one participant can be seen in **Fig 2A** (pre-fatigue) and **Fig 2B** (fatigued).

**Table 2:** Sacral marker trajectory measures pre-fatigue and fatigued represented as mean (SD), along with pairwise comparisons for mean difference and 95% confidence limits of the difference.

Measure	Axis	Pre-fatigue(SD)	Fatigued(SD)	Mean-change[95% CI]	P Value
**Trajectory displacement (cm)**	**VT**	10.72 (1.32)	10.97 (1.44)	0.25 [-0.28, 0.79]	0.33
	**ML**	3.83 (1.36)	4.52 (1.39)	0.70 [0.09,1.10]	**0.002**
	**AP**	7.09 (2.17)	8.54 (2.55)	1.44 [1.14, 1.28]	**<0.001** ^**#**^
**Trajectory range (cm)**	**VT**	13.04 (1.71)	12.95 (1.58)	-0.09 [-0.59, 0.51]	0.76
	**ML**	7.61 (2.09)	9.11 (2.50)	1.50 [0.40,2.59]	**0.006** ^**#**^
	**AP**	9.92 (2.53)	11.73 (3.96)	1.81 [0.60, 3.02]	**0.005**

VT: vertical, ML: mediolateral, AP: anteroposterior

^#^ Log base 10 transformed data

The effect of running fatigue on trunk acceleration variables can be seen in **Table 3**. RMS values increased significantly for all three axes with fatigue. In contrast, the RATIO of acceleration RMS increased in the mediolateral and anteroposterior directions but did not change in the vertical direction. A typical example of horizontal plane accelerations can be seen for the same aforementioned participant in **Fig 2C** (pre-fatigue) and **Fig 2D** (fatigued). Step regularity of accelerations decreased in the anteroposterior direction. No significant changes were detected for stride regularity of accelerations in any axis, or step frequency. Sample entropy of accelerations increased significantly in the anteroposterior direction.

**Table 3:** Trunk accelerometry measures pre-fatigue and fatigued represented as mean (SD), along with pairwise comparisons for mean difference and 95% confidence limits of the difference.

Measure	Axis	Pre-fatigue(SD)	Fatigued(SD)	Mean-change[95% CI]	P Value
**Acceleration RMS (g)**	**VT**	1.39 (0.22)	1.48 (0.21)	0.09 [0.03, 0.15]	**0.007**
	**ML**	0.52 (0.15)	0.64 (0.15)	0.12 [0.08, 0.17]	**<0.001**
	**AP**	0.51 (0.18)	0.61 (0.20)	0.11 [0.06, 0.15]	**< 0.001**
**Ratio of acceleration RMS (a.u)**	**VT**	1.11 (0.10)	1.08 (0.09)	-0.03 [-0.05, -0.01]	0.18^#^
	**ML**	0.41 (0.10)	0.46 (0.08)	0.05 [0.01, 0.09)]	**0.01**
	**AP**	0.39 (0.07)	0.44 (0.10)	0.05 [0.02, 0.09]	**0.01**
**Step regularity (a.u)**	**VT**	0.88 (0.07)	0.87 (0.05)	-0.01 [-0.05, 0.03]	0.56
	**ML**	0.54 (0.16)	0.47 (0.17)	-0.04 [-0.12, 0.04]	0.28
	**AP**	0.57 (0.11)	0.45 (0.18)	-0.12 [-0.18, -0.07]	**< 0.001**
**Step frequency (steps.min** ^**-1**^ **)**	**VT**	162.44 (7.54)	162.88 (8.15)	0.44 [-2.29,3.17]	0.74
**Stride regularity (a.u)**	**VT**	0.87 (0.08)	0.86 (0.05)	-0.01 [-0.05, 0.03]	0.58
	**ML**	0.69 (0.11)	0.70 (0.10)	0.02 [-0.03, 0.07]	0.42
	**AP**	0.65 (0.13)	0.62 (0.15)	-0.03 [-0.09, 0.02]	0.21
**Sample entropy (a.u)**	**VT**	0.17 (0.03)	0.18 (0.03)	0.01 [0.00, 0.02]	0.07
	**ML**	0.43 (0.08)	0.45 (0.09)	0.02 [-0.02, 0.06]	0.37
	**AP**	0.42 (0.11)	0.48 (0.12)	0.06 [0.03, 0.08]	**<0.001**

VT: vertical, ML: mediolateral, AP: anteroposterior

^#^ Based on non-parametric Friedman test

## Discussion

The purpose of the current study was to detect directional changes in dynamic CoM motion in relation to running-induced fatigue based on tri-axial accelerometry. We focused on extracting a select number of measures from a single trunk-mounted accelerometer during speed controlled running until volitional fatigue. Furthermore, the trunk accelerometry measures used in this study were chosen on the basis that 1) they have previously been used to successfully detect limitations in walking gait function, and 2) were calculated relatively easily that avoided algorithms for accurate peak detection or stride-to-stride partitioning. In support of our hypothesis, our main findings indicate that running-induced fatigue resulted in a higher contribution of variability in horizontal plane trunk accelerations, as evidenced by higher mediolateral and anteroposterior ratios of acceleration RMS. In partial support of our hypothesis, only anteroposterior acceleration patterns became less regular and less predictable once running fatigue was induced, as supported by lower step regularity and larger sample entropy values.

Running-induced fatigue greatly influenced the variability of horizontal plane trunk accelerations. This increase was expected, since running-induced fatigue may introduce horizontal compensations in trunk kinematics [11] that may manifest in increased variability of trunk acceleration signals. Additionally, the increase in mediolateral CoM displacements recorded from the sacral marker trajectory confirmed that our fatigue protocol induced greater mediolateral motion of the CoM. Increased trunk acceleration variability mediolaterally indicates an increase in postural sway and greater lateral trunk motion [20,24], which may translate to higher mediolateral impulses during ground contact and expose soft tissue structures to higher strain rates [38]. Le Bris et al., [4] similarly found running-fatigue induced increases in mediolateral trunk accelerations when their runners underwent a highly intensive track running protocol to exhaustion. These researchers [4] speculated that excessive accelerations in the mediolateral direction represents a loss of coordination, with an increase in energy expenditure that is not useful for propulsion. Albeit mediolateral motions being relatively smaller in magnitude compared to the vertical, if reduced, they could act as an important mechanism to facilitate balance control and minimize the energetic cost of running [13,14]. Interestingly, untrained runners have exhibited a higher proportion of horizontal plane (mediolateral and anteroposterior) accelerations of the trunk compared to trained runners [19].

Running-induced fatigue did not influence the step or stride regularity measures except for the anteroposterior step regularity. Step regularity has more recently been used as a symmetry index [23] that represents a correlation of trunk accelerations between left and right steps. It is, therefore, possible that less regular anteroposterior step regularity detected during fatigue may resemble asymmetrical breaking and propulsive phases between left and right running steps. In line with previous investigations of walking gait [10], trunk accelerations were least regular in the horizontal plane. Highly regular vertical accelerations could partially be explained by the fact that the vertical direction of gait is continuously constrained to gravity [39] and thus less subjected to system “noise”.

Sample entropy of trunk accelerations was assessed to gain insight in the non-linearity of the acceleration waveforms. Sample entropy is a useful measure, since it examines every time point of the time-series being analyzed. To the best of our knowledge, sample entropy has never been used to elucidate on running movement patterns. We found that sample entropy values for trunk acceleration patterns were higher and thus less predictable in all three axes when runners were fatigued, although only significant for the anteroposterior direction. This may be partially substantiated by the findings of Meardon et al., [7] who found that runners’ stride times become more unpredictable as they reached exhaustion. According to the “loss of complexity hypothesis”, lower sample entropy values relate to a more predictable physiological time series that is often represented by frailty, disability or disease due to an unhealthy biological steady-state [25,40]. In line with this hypothesis, human locomotion studies have reported lower sample entropy values of populations with pathologically-related walking gait [35,36]. Therefore, we hypothesize that our more variable and less predictable anteroposterior time-series (higher sample entropy) reveals an overall protective neuromuscular CoM control to preserve musculoskeletal structures and avoid pain [3] due to fatigue. A theory which therefore remains untested is whether runners with a history of overuse injuries fail to reduce regularity and predictability of trunk acceleration patterns when in a fatigued state.

The indoor treadmill protocol we selected was chosen on the basis of selecting a controlled steady state environment. An important question to ask is whether certain measures that changed with fatigue, such as anteroposterior step regularity for example would change even more in an overground situation. Firstly, the speed of running is naturally more variable overground than compared to treadmill running, even under controlled conditions [41]. Secondly, we noticed that in a fatigued state, our runners were struggling to maintain anterior positioning on the treadmill belt at their individually constant speed. Therefore, had the fatigue protocol been performed overground, we expect that our runners would have slowed down as a protective means [42], resulting in more variable trunk accelerations anteroposteriorly. Thus even greater changes in anteroposterior step regularity could be expected. To improve generalizability, further steps are aimed at determining the robustness of the current accelerometry measures to running related fatigue in self-paced outdoor environments, where runners would typically counter fatigue by reducing their running speed [42]. Therein lies large potential to monitor the biomechanical-aspects of running, with the aim of detecting early onset of excessive variability or irregularities of horizontal plane accelerations due to fatigue [4]. In conclusion, our findings indicate that a single trunk-mounted accelerometer can be used to detect deviations in dynamic CoM motion induced by running-fatigue. In light of our results, running-induced fatigue might reveal itself in a greater contribution of variability in horizontal plane trunk accelerations, and anteroposterior trunk accelerations that are less regular from step-to-step and less predictable.
